# A method for early detection and identification of fungal contamination of building materials using e-nose

**DOI:** 10.1371/journal.pone.0215179

**Published:** 2019-04-09

**Authors:** Zbigniew Suchorab, Magdalena Frąc, Łukasz Guz, Karolina Oszust, Grzegorz Łagód, Agata Gryta, Nina Bilińska-Wielgus, Jacek Czerwiński

**Affiliations:** 1 Faculty of Environmental Engineering, Lublin University of Technology, Lublin, Poland; 2 Institute of Agrophysics, Polish Academy of Sciences, Lublin, Poland; Georg-August-Universitat Gottingen, GERMANY

## Abstract

The aim of the study was to develop a method for early detection and identification of fungal contamination of building materials using an electronic nose. Therefore, the laboratory experiments based on the analysis of the air in the vicinity of fungal isolates potentially found in the building materials were performed. The results revealed that the employed gas sensors array consisting of MOS-type sensors enables the detection of the differences among the examined samples of fungi and distinguishing between the non-contaminated and contaminated samples, shortly after fungal contamination occurs. Electronic nose readouts were analysed using Principal Component Analysis and the results were verified with standard chromatographic analysis by means of SPME-GC/MS method, which proved that gas sensors array can be applied for early detection of fungal contamination.

## Introduction

Microorganisms inhabit different types of biological niches on the Earth, due to their adaptability to varying environmental conditions. It is well-known that microorganisms cause deterioration of various materials. Microscopic fungi induce biological corrosion of construction materials, as well as rotting of food. Fungal metabolic products can be detrimental to the human health due to their toxicity. Therefore, a quick preliminary assessment of the occurrence of fungi in buildings is recommended and vital for maintaining healthy population, as well as high quality of building materials and indoor air [1].

Until now, the detection and identification of fungal contamination of buildings was based on culture-dependent methods. Such approach was time-consuming and required specialized laboratory equipment. In spite of many advantages, these methods provide limited information because they only identify viable organisms capable of growth under standard laboratory conditions. Majority of microorganisms in natural samples cannot be cultured according to standard procedures and the culture-dependent methods provide limited information on the biodiversity of microorganisms thriving on objects. On the other hand, molecular methods are essential tools for analysing microbial communities structure [2]. However, they are still expensive, require specialized knowledge and are performed *ex situ*, which means that the samples of contaminated materials should be collected and examined in laboratory. In order to improve the effectiveness of raw material management, more rapid, early detection techniques are required [3,4]. The development of sensor-based devices over the last few years, presents an opportunity to use these tools for the purpose of a more effective fungal detection in contaminated materials.

### Problems of mould fungi in buildings

According to World Health Organization (WHO), the indoor air quality has greater impact on human health than the outdoor air. This is due to the fact that people in developed countries spend up to 70% of their time indoors [5]. Apart from that, numerous studies conducted in Poland and other countries confirm that the concentrations of pollutants are significantly higher in the indoor than the outdoor environment [6].

Since the advent of energy-saving construction technologies, architects focus on methods which enable to contain heat in buildings. This is achieved by means of airtight windows and doors. Increasingly thicker insulation layers are being built in walls, protecting them from the influence of wind, but also from vapour exfiltration, which is considered as a negative phenomenon. Houses are airtight to a much greater degree than in the past, which contributes to an increased concentration of air pollutants. Each day, people are being exposed to dust, pollen, mould spores, chemical contaminants released by building materials, as well as interior furnishings and equipment. Moreover, people exhale carbon dioxide and water vapour while doing household chores–washing, cooking, bathing, etc.–which constitute a significant source of humidity [7].

It is difficult to unequivocally determine which pollutants merely cause discomfort of inhabitants and which may lead to an illness. Sick Building Syndrome (SBS) has been a major topic for many years and is the most distinctive result of inadequate indoor air quality. Its characteristic symptoms include conjunctivitis, asthma, chronic laryngitis and bronchitis, migraines, irritability, difficulties in concentrating leading to the loss of motivation and engagement at work and drop in efficiency [8].

Indoor air quality is influenced by such factors as the thermal comfort (subjective feeling related to temperature and humidity), odours (olfactory substances) and other, odour-free substances. Significant amounts of these substances may be produced by various fungi genera. Fungal cultures develop mainly in damp building materials, which constitute source of nutrition for these microorganisms. Atmospheric air always contains a certain amount of microorganisms, including fungal spores. It must be noted that under normal conditions, the amount and types of microorganisms found indoors and outdoors is similar. On the other hand, it is known that in damp buildings both these aspects are completely different [8].

Most favourable humidity and temperature ranges vary depending on the fungi genus or substrate. However, it may be assumed that the relative air humidity over 75% creates optimal conditions for the development of mould fungi, thus contributing to the contamination in buildings [9].

Nowadays materials which are of biological origin like hemp, flax, sunflower or wood become more common [10–13]. Their application is additionally increased by the popularity of sustainable building [14] idea which imposes to use eco-friendly building materials, which may reduce the environmental impact made by the building sector and enable to minimize CO_2_ emissions. Unfortunately, plant origin building composites have many nutritious elements attractive for microorganisms, that is why they are significantly prone to fungal contamination.

According to [15] the concentration of fungal spores in the contaminated buildings, expressed in colony forming units (cfu) exceeds 1000 cfu/m^3^, while the permissible amount equals 50 cfu/m^3^ in the case of mould fungi and 150 cfu/m^3^ in the case of mixed genera–except pathogens. With *Cladosporium* and *Alternaria*, the permissible level amounts to 300 cfu/m^3^, while the presence of *Aspergillus fumigatus* and *Stachybotrys atra* is inadmissible [16]. According to Piotrowska and Żakowska [17], the dominant genera in buildings include: *Aspergillus versicolor*, *Penicillium chrysogenum* and *Cladosporium cladosporoides*. In moderate climate countries, the most dangerous genus is *Serpula lacrymans*. It infects hardwood and softwood, as well as wood-like materials. This fungus has a preference for 30% wood humidity and the temperature of 23°C. Wood stricken by *S*. *lacrymans* loses 7% of mass per month, while its strength parameters drop by 20–50% [18].

### Influence of mould on indoor air composition

Fungal contamination of the indoor environment is mainly caused by the presence of Microbial Volatile Organic Compounds (MVOCs), which constitute a sub-group of Volatile Organic Compounds (VOCs), mycotoxins, fungal spores and fragments of mycelia. MVOC-type compounds with a distinctive smell of mould indicate the presence of filamentous fungi inside buildings. As the microorganisms develop, they release these compounds, which may cause various health problems [19]. On the other hand, mycotoxins are toxic substances produced by fungi. Toxins produced by *Stachybotrys*, *Fusarium* and *Aspergillus versicolor* are considered the most harmful [8,20]. The most toxic mycotoxins produced by mould fungi include aflatoxins (AF), ochratoxin A (OT), zearalenon (ZEN), trichotecenes and fumonisins (F) [8,21–23]. Other toxins produced by mould fungi comprise alcohols, ketones, terpenes, esters, and sulphur compounds, including the following substances [24–27]: Alcohols: 2-methyl-propanol, 3-methyl-1-butanol, 2-metyl-1-butanol, 3-octanol, 1-octen-3-ol, 1-hexanol, 1-pentanol, 2-methyl-isoborneol, geosmin; terpenes and sesquiterpenes: limonene, pinene; ketones: 3-octanone, 2-heptanone, 2-pentanone; furanes: 3-methylfuran; sulphur compounds: dimethyl disulphide; hydrocarbons: alkanes, alkenes, dienes, trienes.

Issues connected with the fungal contamination of building elements resulting in changes of the air quality are extremely important both from the medical and technical standpoint. Early detection of the substances indicating fungal contaminations may help in selecting appropriate preventive measures and aid in removing the damage from infestation. Renovation of a damp building cannot be carried out without the prior professional mycological evaluation of building structures, interior furnishings and stored materials. Moreover, identification of fungi can indicate how dangerous is the genus being dealt with.

### Detection techniques of fungal contamination

Traditional methods of enumerating fungal contaminants require a lot of time and manual labour. In order to improve the effectiveness of raw material management, more rapid, early detection techniques are required. The development of sensor-based devices over the last few years, presents an opportunity to use these tools for the purpose of a more effective identification of fungi in contaminated materials.

Methods which involve monitoring of the selected markers, e.g. mycotoxins (HPLC-MS/MS method), require a complicated sample preparation process. Therefore, SPME-GC-MS or Selected Ion Flow Tube-Mass Spectrometry (SIFT-MS) methods, which enable the analysis of volatile fungi metabolites, seem to be adequate in this case [28].

Many genera of fungi produce a specific range of volatiles [29,30]. This profile (gas fingerprint) could be utilized for early detection of fungal contamination of stricken materials. Electronic or optoelectronic nose technology has been previously used in many branches: food [31,32], medical [33–36] or environmental industries [37,38]. Therefore, the possibility of identifying fungal contamination of samples and their genera, carried out on the basis of headspace analysis is relevant for environmental or civil engineering, both from the scientific and practical standpoint. Such studies are important especially in the early stages of fungal development, when their presence is not yet visible, the concentration of spores is low, and there is no obvious development of mycelia. Therefore, the application of electronic nose based on MOS (metal oxide semiconductors) gas sensors array for a rapid evaluation and identification of samples contaminated with various fungi genera–including the ones usually found in building seems to be a promising approach.

The aim of the conducted studies was to determine the possibility of utilizing electronic nose with MOS gas sensors array for the detection and identification of the genus of fungi in microbiologically contaminated building materials.

## Materials and methods

Experiments involved performing the analysis of the air in the vicinity of fungi potentially found in the human environment as moulds in the residential buildings. The study included testing of 10 fungal strains isolated from contaminated building materials belong to the following genera: *Penicillium* sp. (G5/15, G6/15, G11/15, G13/15a, G14/15), *Aspergillus* (G8/15, G9/15), *Acremonium* sp. (G7/15), *Paecilomyces* sp. (G10/15), *Cladosporium* sp. (G13/15). The fungal isolates used in the study were selected based on their dominance in tested buildings materials. Representatives of *Penicillium* and *Aspergillus* were dominant in fungal community, whereas *Acremonium*, *Paecilomyces* and *Cladosporium* were represented only by single isolates.

### Materials

Mould fungi were isolated from the samples of materials subjected to capillary action. The samples were prepared from an experimental construction material, which was a composite of hemp and flax shives, as well as sand and calcium-based cementing material, modified with mineral additives. This composite is utilized in civil engineering as a light, ecological construction material which is used mainly for insulation and construction of walls. Its parameters and applications were presented in the following works [10,11]. Fungal contamination resulted from the experiments on humidity and capillary action performed on the sample. Hence, the sample was exposed to high humidity, which was conducive to the development of fungi. Fungal strains were isolated 45 days after the initial contact of samples with water and achieved 56% of volumetric moisture, which closely resembles the state of full saturation.

Ten strains of mould isolated from building materials were identified through macroscopy and microscopy observations. Afterwards, they were classified to the appropriate genera. Pure fungi strains isolated from construction material were cultured on Potato Dextrose Agar (PDA), on 55 mm diameter Petri dishes at 30°C, kept in the dark for 10 days. Then 200 ml of PDB medium in 1l flask was inoculated by spores harvested with a sterile swab from the surface of two Petri dishes. Inoculated PDB medium was incubated for 360h. Moulds and the control samples during the experiments were stored in a Microbiological Incubator (Binder, BD240) at the temperature of 25°C. Measurements were performed after 1, 3, 6, 12, 24, 48, 72, 120, 192, 288 and 360 hours of incubation, by means of an e-nose. In the same time visual evaluation of samples was conducted. The experiments were carried out in two biological replications for each sample.

### Methods

#### Electronic nose and test stand description

The main element of the e-nose comprised a gas sensors array and the appropriate set of converters. The array consists of eight MOS-type (metal oxide semiconductor) gas sensors (TGS Figaro, USA). The following sensors were used for that purpose: TGS2600-B00, TGS2602-B00, TGS2610-C00, TGS2610-D00, TGS2611-C00, TGS2611-E00, TGS2612-D00, TGS2620-C00. These sensors are widely-applied owing to their low cost and high reliability [39,40]. Moreover, two additional sensors were installed in order to measure the environmental conditions, i.e. DS18B20 temperature sensor (Maxim-Dallas, USA) and HIH-4000 relative humidity sensor (Honeywell, USA). In the case of the presented experiment, their significance was relatively low, due to the stable conditions of the conducted measurement. Nevertheless, they enabled to verify the stability of the experiment conditions. The sensors array was located in a covered, temperature-stabilized chamber in front of the e-nose.

Sampling was carried out by means of a polyamide tube, mounted with a quick disconnect coupling to a cover of a gas sensors chamber. External diameter of the hose was equal to 4 mm, its internal diameter was 2 mm, while the length was 50 cm; the end was fitted with a 1.2×40 mm needle. The required flow of gas sample was provided by FM1101 F6V membrane micro-pump (Fürgut GmbH, Germany), which was built in the e-nose. The measured nominal flow amounted to 100 ml/min. During the research, the flask plug was removed and replaced with sterile aluminium foil. While taking the measurements, the foil was penetrated with a needle; the mouth of the flask was sterilized with burner flame afterwards. Maintaining sterile conditions and instruments was necessary in order to prevent the contamination of individual samples.

**Fig 1:**
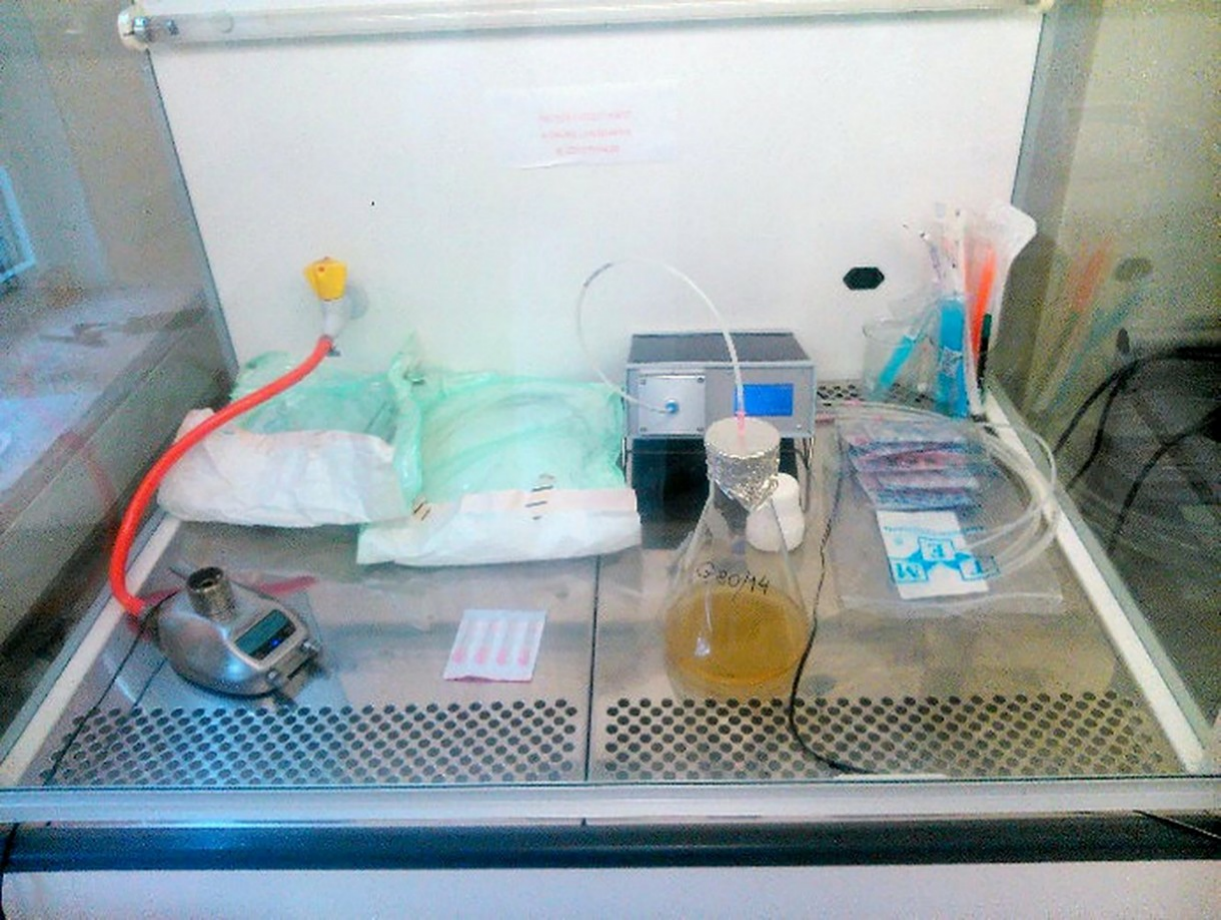
Photograph of the test stand.

The measurements were carried out in Lamil Plus 10 laminar chamber (Karstulan Metalli Oy, Finland) (Fig 1). The air supplied to the chamber was purified with HEPA filters; its flow rate inside the chamber was equal to 0.4 m/s.

Before each measurement session, the sensors array was heated until the temperature in the sensor chamber was stabilized. The array and its equipment were exposed to UV light after their placement in the laminar chamber. Gas sensors were flushed for 10 minutes with air from the laminar chamber. Afterwards, the measurements were performed for control groups (non-inoculated) and experimental ones (inoculated with individual groups/genera of fungi). Each measurement took 7 minutes and was divided into two phases: 2 minutes of treatment with clean air from the laminar chamber and 5 minutes of sampling the air from the laboratory flask containing the samples. An attempt to devise a quick method for evaluating the risk of fungal contamination provides an additional justification for a 5-minute measurement period. Data acquisition from all sensors took place in 1-second intervals. Large amount of data obtained during the measurements of each flask allowed the preparation of a broad statistical analysis and tracking a potential signal drift. The data collected by sensors array in the last 30 seconds of measurement was considered for further analysis.

The measurements yielded a multi-dimensional set of data. Individual variables (readings from each sensor) were standardized. Relative resistance was determined according to the following formula:

$$R_{R}=R_{S}/R_{O}$$ (1)

where: *R*_*S*_−sensor resistance [kΩ] during the measurement of gas phase, *R*_*O*_−sensor resistance [kΩ] in clean air.

The Principal Component Analysis (PCA) was performed by means of covariance matrix. The comparison involved the two main components with the highest values of eigenvectors, which enabled to describe the most differences within the set of data. All the presented data were transformed according to the same matrix of eigenvectors.

#### Chromatographic analysis

Experiments involving the obtained compounds (metabolites) were also conducted through the chromatographic analysis, by means of SPME method. Isolating and enriching the analytes were carried out with the following fibres (Supelco, USA): non-polar fibre (100 μm PDMS), moderately polar fibre (SDVB 70/30). The fibres were exposed for 15 minutes. Proper analysis was carried out by means of a gas chromatograph, coupled with Trace Ultra-Polars Q mass spectrometer (Thermo, USA).

The parameters of chromatographic analyses were the following: fibre desorption time– 1 minute, at the temperature of the injector equal to 250°C, PTV injector (Programmable Temperature Vaporizer) operating in constant temperature mode with 2 min split opening time, as a carrier gas helium, purity of 99.9996 (BOC, Poland) was used; carrier gas linear speed was 0.40 m/s–optimal for the diameter of the applied column (RTx-5 MS, 60 m × 0.25 mm, coated with 0.25 μm-thick 95% PDMS and 5% PDPhS stationary phase, Restek, USA). Oven temperature programme– 60°C (1 min), with the increase rate of 10°C/min, up to 300°C (10 min) was applied. Transfer line temperature was set to 250°C. Applied MS operating mode was full scan monitoring in the mass range of 50–350 m/z; ionization potential: 70 eV; applied collision gas was helium (0.3 ml/min). Data acquisition and post-processing was performed by Excalibur v. 2.02. Spectral libraries applied for identification–NIST 2005 and Wiley 8^th^ Edition. Similar methodology was applied in the following researches [41,42].

## Results

The conducted research is divided into the following stages: (i) differentiation between contaminated and non-contaminated samples, (ii) identification of individual genera of fungi, (iii) chromatographic analyses of fungal metabolites.

### Differentiation between contaminated and non-contaminated samples

The basic and the first approaches of e-nose application for fungal contamination detection were mainly focused on determination of the contamination status (contaminated or not-contaminated). This stage of the study was designed to investigate the performance of e-nose in the detection and discrimination of samples infected by fungi extracted from building materials in the early stage after infection. The fast detection of differences in fungal contaminated and not-contaminated samples is critical in monitoring of the building.

Principal Component Analysis (PCA) method was applied for data post processing. During the PCA analyses, two main components were determined: PC1, which accounts for 80.7% of variance and PC2, which accounts for 10.9% of information concerning the initial set of data.

**Fig 2:**
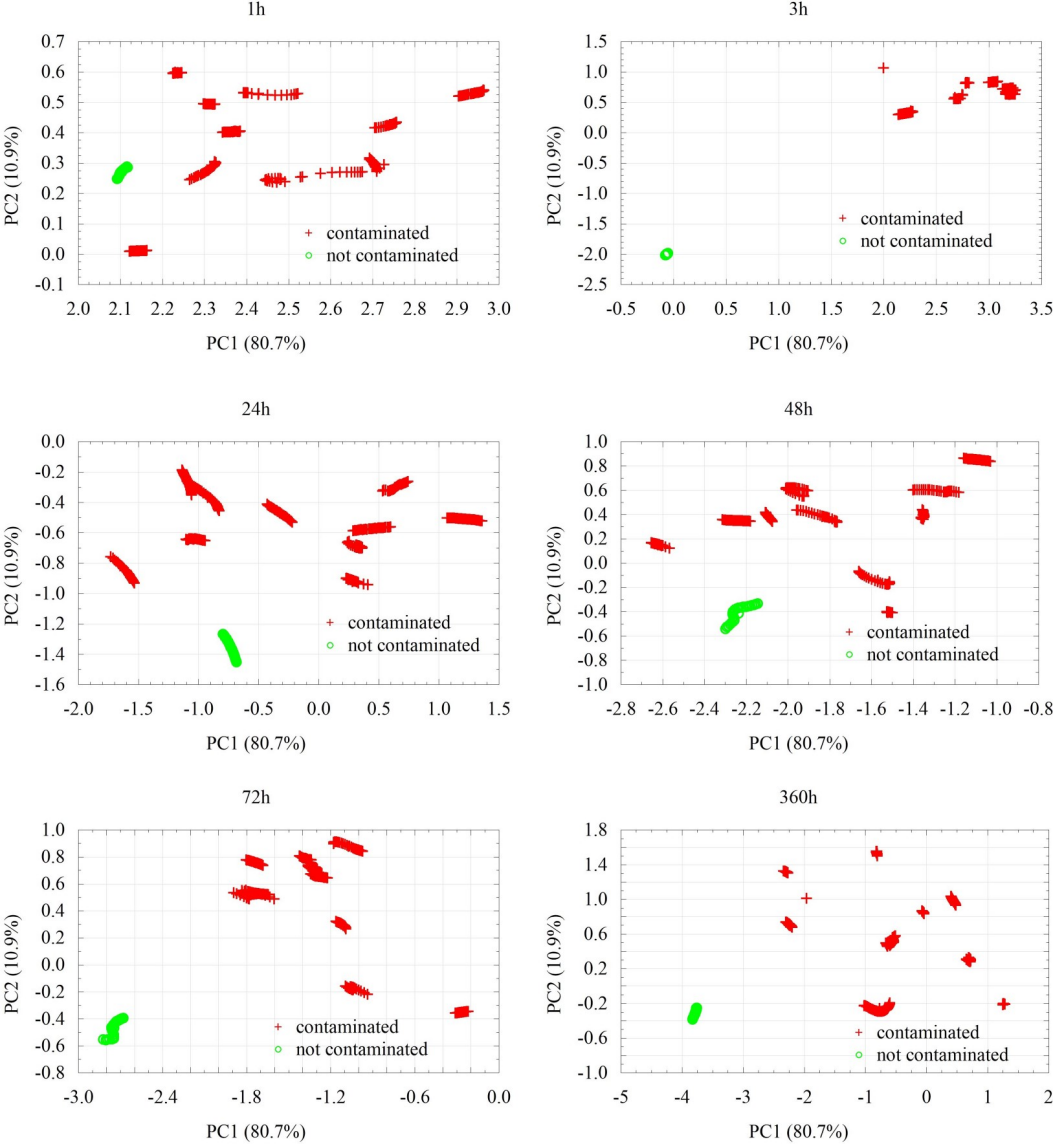
PCA results for contaminated and non-contaminated samples with different time of growth, given in hours.

Fig 2. presents the division of considered samples depending on the presence or lack of fungal contamination. The samples contaminated with fungi were marked with red, whereas the non-contaminated control samples were marked with green. The figure presents the key set of data obtained earlier, from the moment the substrate was contaminated. It seems that the greatest difference between the non-contaminated and contaminated samples is observed in the third hour. However, in the case of contaminated samples, it would be difficult to identify the genus of fungi by then. Although these differences are better visible starting from the 24^th^ hour which could allow for fungi identification, the differences between the contaminated samples and non-contaminated control sample begin to fade away. Such tendency is visible till end of experiment (at 360^th^ hour).

Visual analyses enabled to detect fungal growth of the examined samples after two or more days after inoculation. In general it was noticed that most mould identified as *Penicillium* sp. and *Paecilomyces* sp. appeared at the surface of the PDB medium after 72 hours since inoculation when fungi belonging to *Aspergillus* sp. and *Acremonium* sp. was noticed after 120 hours since inoculation.

### Identification of individual genera of fungi

PCA technique was also applied for fungi genera identification. During the PCA analyses, two main components were determined: PC1, which accounts for 80.7% of variance and PC2, which accounts for 10.9% of information concerning the initial set of data.

**Fig 3:**
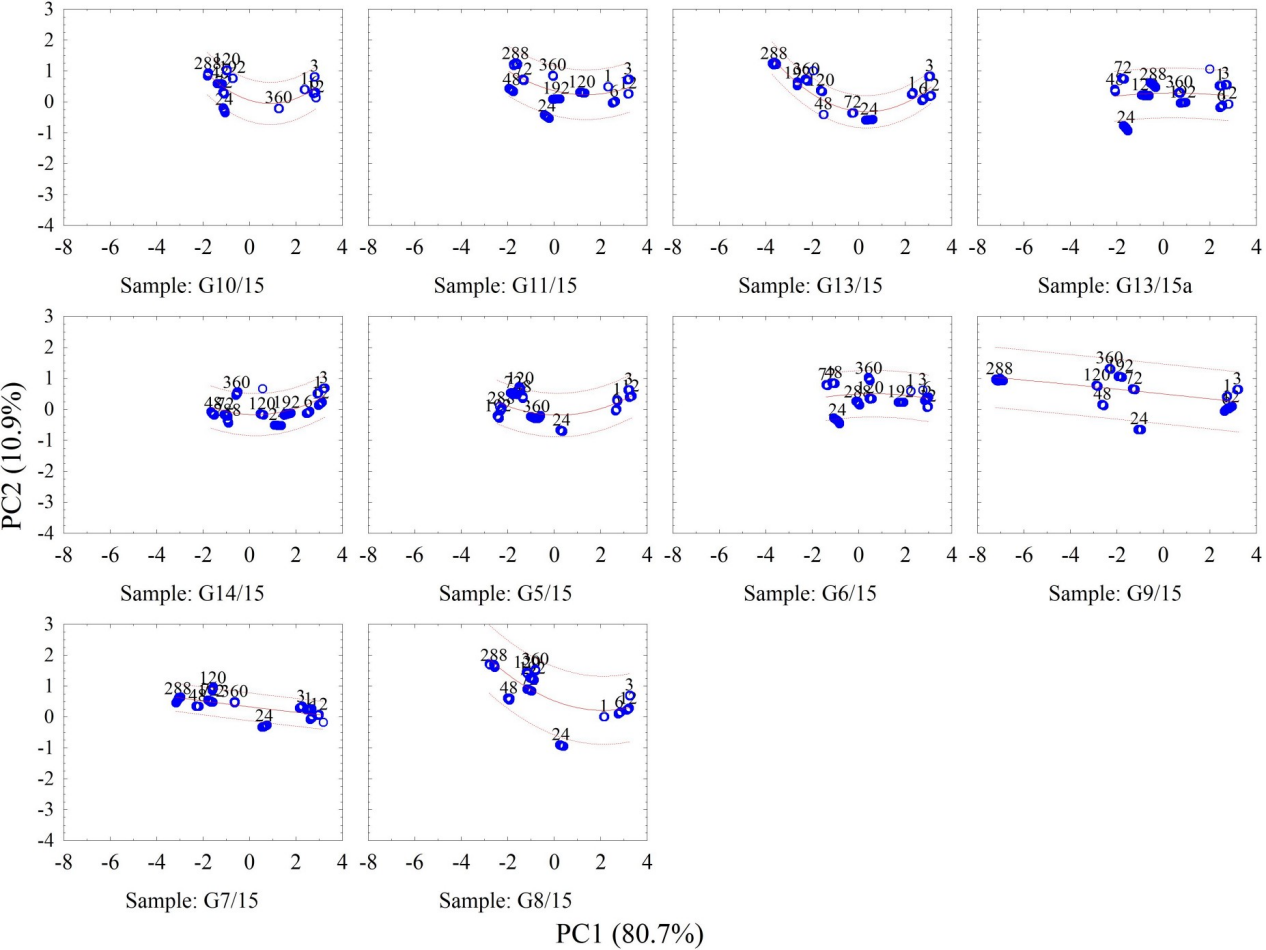
PCA results concerning changes for individual fungi isolates, occurring in consecutive hours of the experiment–including the trajectories of changes and confidence interval 0.95.

Fig 3 presents the results of PCA, showing the array signal in consecutive hours (from the beginning of the research) for individual fungal genera. According to the graphs, the composition of air sampled from the test tube undergoes changes in consecutive measurement series, which is visible as a group of dots in respective areas of the graph. Generally, all the changes which occur in the sensors array signal with time are characterized with the same sense in relation to PC1. The higher concentration of tested fungal metabolic products above the medium caused more significant movement of points towards the negative values of PC1. In order to enable the identification of fungi with a satisfactory accuracy, at least 3 measurements should be performed in appropriate time intervals (every 12 or 24 hours); afterwards, the direction, sense, and trajectory of array signal changes in time can be taken into consideration.

As can be seen in Fig 3., initially, the array signal dots for all samples are located close to each other in the two-dimensional space of the same scale. However, the changes of array signal that occur with time–starting from the moment of contamination–differentiate the samples to a significant degree. While analysing the individual trajectories, a certain tendency can be observed–the signals after PCA analysis form two groups (this is more visible in case of some samples and less for others). The first one is located at the right side of the graphs. It corresponds to the readings of samples from the first day of the experiment (1, 3, 6, and 12 hours) and signifies low metabolite levels detected by the sensors. The other set groups the remaining readings from the following measurement sessions (from 24 to 360 h); however, in the case of most samples, the coordinates in the set obtained after PCA which are located the furthest from the measurements performed in the first day, are displayed by the array signals for the sample analysed in the 288^th^ hour.

On the other hand, the coordinates for the measurements performed in the 360^th^ hour are closer to the readings from the first day. This can be attributed to the loss of metabolic activity of fungi, resulting from the exhaustion of nutrients in the medium.

**Fig 4:**
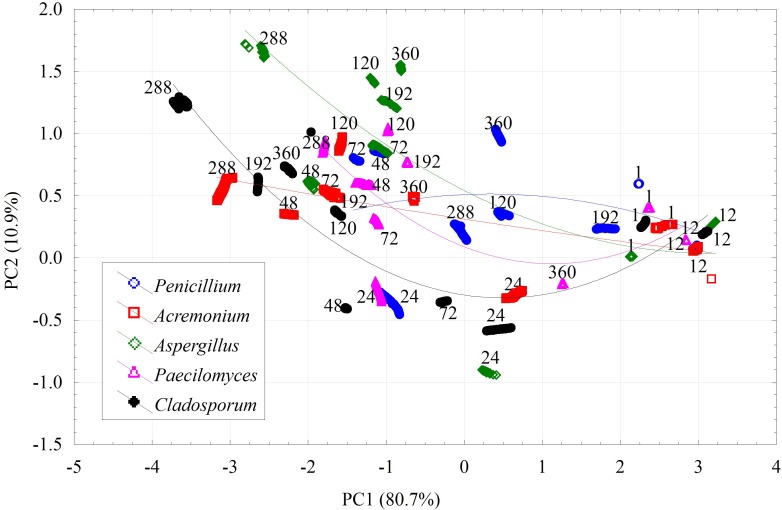
PCA results concerning changes in the sensors array signals for the selected fungi isolates, occurring in consecutive hours–counting from the beginning of the experiment.

Fig 4. presents the PCA results concerning changes of sensors array signal in consecutive hours–from the beginning of the experiment–for selected fungi isolates: *Penicillium* sp. (G6/15), *Acremonium* sp. (G7/15), *Aspergillus* sp. (G8/15), *Paecilomyces* sp. (G10/15) and *Cladosporium* sp. (G13/15). The choice was made on the basis of the analysis of Fig 3, where these samples were characterized with a different trajectory of signal changes. Considering solely the two-dimensional space of main components, these isolates can be identified with a satisfactory accuracy after the 120^th^ hour, albeit the differences in signals are already easily detected after 24 hours. Similarly as in the previous figure, the greatest differences in the signal read from the sensors array are observed in the 288^th^ hour from the beginning of the experiment.

**Fig 5:**
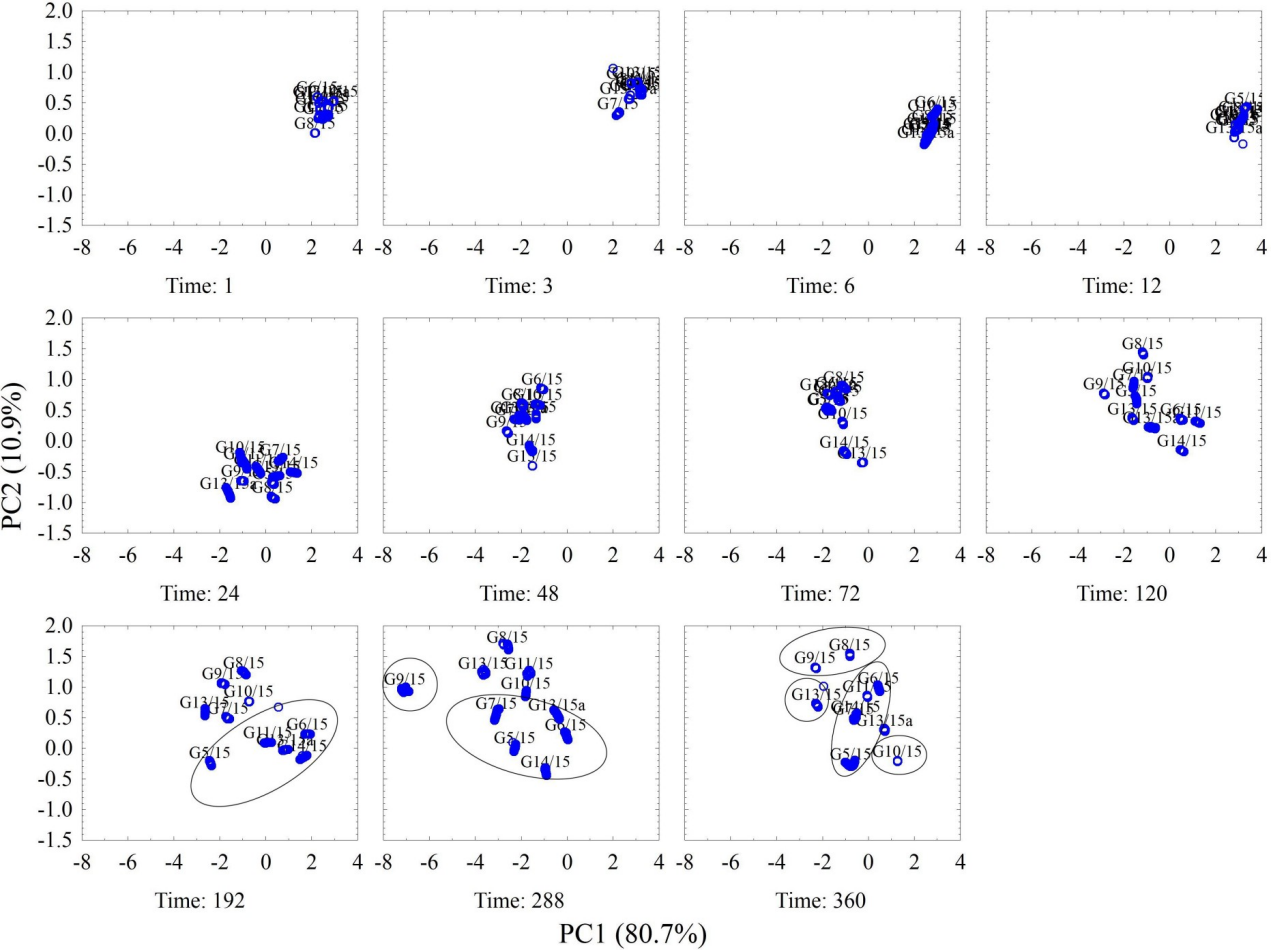
Results of PCA for all fungi isolates divided into individual hours, from the beginning of the experiment.

The identification of the genera of fungi is easier when the exact growth time is known. Fig 5 shows the results of PCA for all fungi isolates, split into individual measurement series indicated as hours, starting from the beginning of the experiment. In the first 12 hours, there are no visible changes, when using the fixed, common scale of reference. Starting from the 24^th^ hour, the dot clusters of individual fungi are beginning to differentiate. The greatest differences were observed in the 288^th^ hour. In the 360^th^ hour, one can observe a reversal in the direction of cluster movement for individual fungi isolates, however the results analysis allowed to cluster the fungal strains according to the genera.

The separation of *Penicillium* sp. (G5/15, G6/15, G13/15a and G14/15) from the other fungal genera was visible from 192^th^ incubation hour. Differentiation between particular fungal genera was clearly marked at the 288^th^ hour of experiment, especially for *Aspergillus* sp. (G9/15) and *Penicillium* sp. (G5/15, G6/15, G13/15a and G14/15) clustered together with *Acremonium* sp. (G7/15). At 360^th^ hour of experiment the mostly tested fungi clustered in separate groups according to the genera. First the biggest group included *Penicillium* sp. (G5/15, G6/15, G11/15, G13/15a and G14/15) and *Acremonium* sp. (G7/15) fungi, the second clustered the *Aspergillus* sp. strains (G8/15, G9/15). Fungal isolates belonged to *Paecilomyces* sp. (G10/15) and *Cladosporium* sp. (G13/15) created independent groups.

### Chromatographic analyses of fungi metabolites

Chromatographic analysis was used as a reference method to compare headspace analyses conducted with an e-nose. The differences in volatile compounds observed in contaminated samples as well as the not-contaminated controls were analysed by GC-MS and head-space–solid phase microextraction (HS-SPME).

In the case of the first two measurements that followed 12 and 24 h after inoculation, all parameters were below 0.5 mg/m^3^, which is considered to be under the detection threshold, and will not be discussed further.

Table 1 contains average values of two samples measurements that were conducted 72, 288 and 360 hours after inoculation, labelled as A, B and C, respectively.

**Table 1:** Data obtained by chromatographic analyses of samples headspace.

Strain number	Measurement	Benzene [mg/m^3^]	Toluene [mg/m^3^]	Ethylbe nzene [mg/m^3^]	Xylenes [mg/m^3^]	Trichloro ethylene [mg/m^3^]	1,4- Dichloro benzene [mg/m^3^]	Methyl or ethyl esters of benzoic acid
G5/15	A		43					
	B		46				26	
	C		37				17	
G6/15	A		51				81	
	B	26	57			17	81	
	C		26				34	
G7/15	A	27	63	26		18	54	
	B	31	82	21		26	42	
	C	11	64			18	42	
G8/15	A		18			22	36	
	B		18			27	43	x
	C		18			22	36	x
G9/15	A		22				92	
	B	44	26				61	x
	C		19				53	x
G10/15	A		56	31			46	
	B		48	24			74	x
	C		53	29			62	x
G11/15	A		44	44	57	43	51	
	B		32	48	49	51	68	
	C		28	26	42	41	29	
G13/15	A		78	32			56	
	B		72	39	18		59	x
	C		41	32			18	x
G13/15a	A		97				43	
	B		127				52	
	C		72				26	
G14/15	A		122				29	
	B		143				42	
	C		46				12	

**Fig 6:**
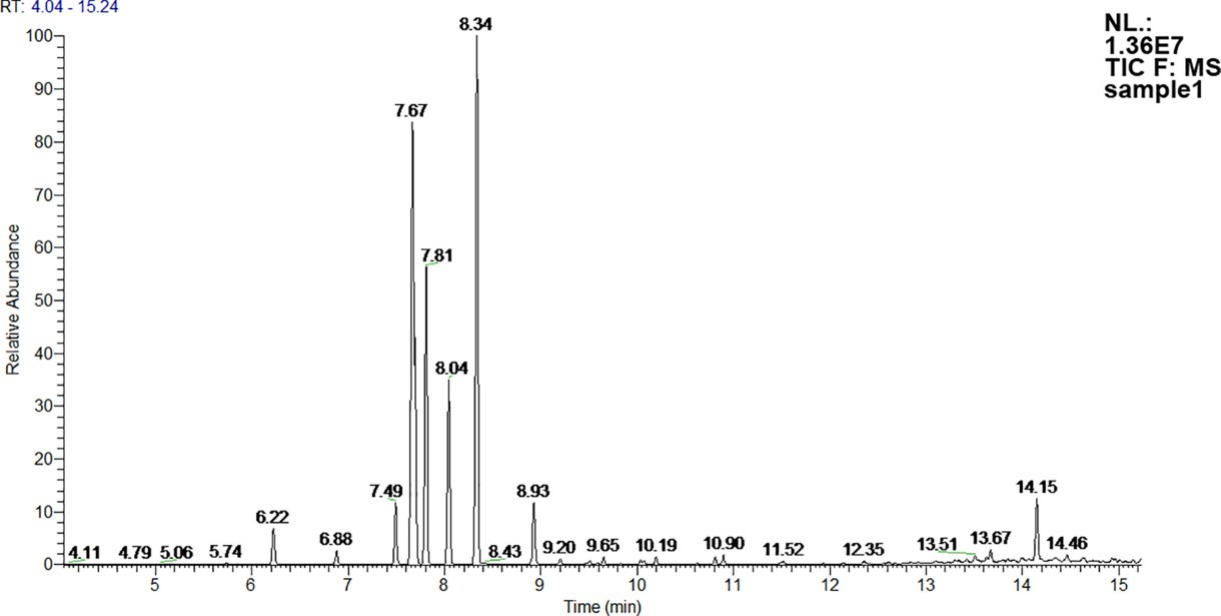
Typical TIC GC-MS chromatogram of metabolites found in the headspace above live fungal culture.

**Fig 7:**
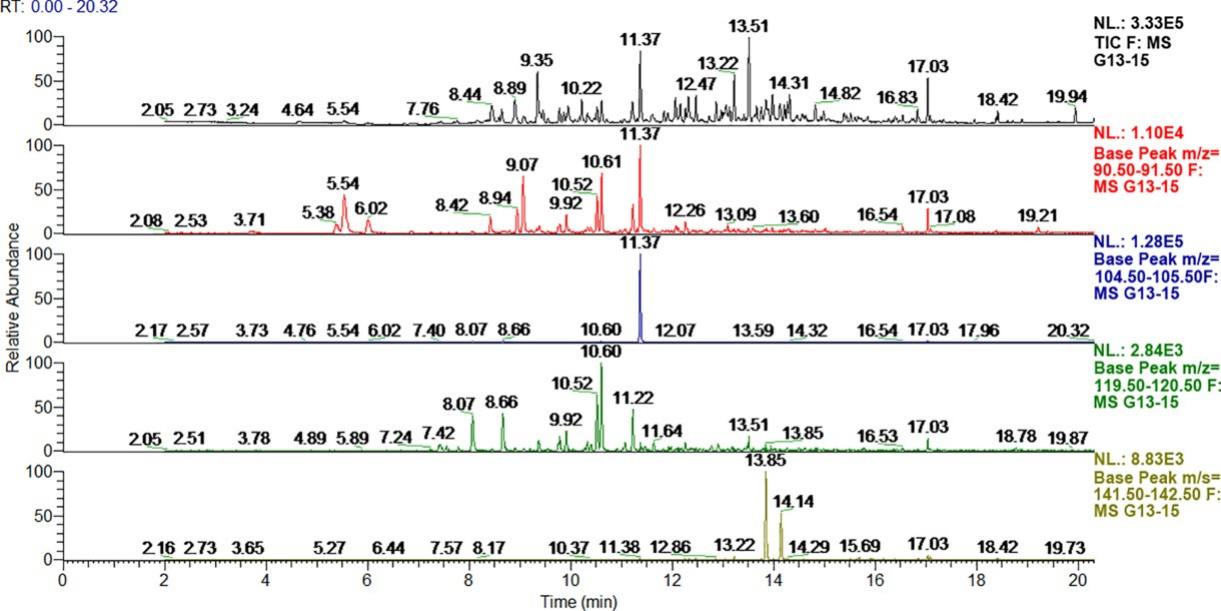
HS-SPME chromatogram of the selected fungus *Cladosporium sp*. (G13/15) with fragmentograms of characteristic compounds: toluene, xylenes, 1,4-dichlorobenzene at measurement (B) after 288 hours since inoculation.

Figs 6 and 7 present the HS-SPME-GC-MS chromatograms of metabolites found in the headspace above living fungal culture. Fig 5. is a typical chromatogram of Total Ion Current (TIC) observed during incubation. Fig 6. contains fragmentograms which present characteristic ions used for determination of the selected compounds.

Determination was conducted with HS-SPME method in order to avoid contamination of the sample with other microorganisms. This method also prevents the introduction of other pollutants. In the case of chromatographic determination, the variability and abundance of metabolites are significant.

The presence of monoaromatic hydrocarbons (toluene) is a characteristic feature of all cultures. Interestingly, halogenated hydrocarbons–especially 1,4-dichlorobenzene–appear as well. It seems that this compound (due to its confirmed presence in almost all cultures) is a valid indicator of fungal infestation.

All samples were also analyzed for methyl and ethyl benzoate, tetrachloroethylene and 1,2-dichlorobenzene; however, they were not detected in significant amounts [42].

It is also worth mentioning, that metabolites can be dependent on the fungal strains found in a given area [42]. The usefulness of this method is questionable, as the highest results are at the limit of detection. Trichloroethylene and benzene were produced especially by *Acremonium* sp. (G7/15), and some *Penicillium* sp. (G6/15, G11/15) fungi. They were not found as a metabolite of the other tested fungi, therefore these compounds can be used as a marker of contamination by mentioned moulds.

It should be underlined here that traditionally applied testing methods, such as GC-MS and HS-SPME, gave information about the increase of the fungi metabolites levels in the measurement (A) conducted after 72 hours since inoculation. In the measurements conducted after 12 and 24 hours since inoculation, the detection thresholds were not exceeded. The highest levels of detected substances produced by fungi were observed in the measurement (B) that took place after 288 hours since inoculation, but it should be noticed that the increase was not significant, sometimes a decrease of levels of detected substances was observed as well. Finally, in the last measurement (C) conducted 360 hours after inoculation, the decrease of detected substances levels, which should be considered as the exhausting of nourishing substances and the decrease of fungi development, is clearly visible. This tendency was also visible during the PCA analyses of the e-nose readouts and was used to define the terminal point of the experiment.

## Discussion

In the traditional approach, the degree of fungal contamination in buildings is expressed as the amount of spores in the air, settling dust, surface or samples collected from construction materials. Therefore, the use of traditional mycological methods is time-consuming and long-lasting. Although many highly-sensitive and precise molecular and immunological methods were developed to fast detection and identification of fungi in the last decade, they are still very expensive and require adequate laboratories, equipment and experienced staff [43]. As a viable alternative, one may employ the method consisting in measuring the concentration of fungal metabolic products in the indoor air, including MVOCs [34].

According to Kuske et al. [24], MVOCs are good indicators of the presence of fungi in buildings. Detection of VOCs by means of an e-nose is faster than the traditionally employed methods. MVOCs analyses enable early detection of fungi, even before spores appear. They even allow to detect fungi which do not produce or release spores at all, as well as the ones which develop in hidden, covered places that are impenetrable for the spores but penetrable for metabolites (MVOCs). In such situation, traditional biological methods of fungi detection would yield negative results, whereas an e-nose is able to track their presence and confirm the fungal contamination.

This was also proved by the results presented in our research. The achieved results confirmed that e-nose analyses detected fungal contamination earlier than chromatography methods. Taking into consideration that it is possible to detect fungal spores in the air applying the traditional techniques of fungal detection after the period of 72–120 hours, MOS sensors matrix combined with PCA analysis enables to ascertain the fact of fungi metabolites presence after 3 hours since inoculation. Visual observation proved fungal growth after 72–120 hours after inoculation. However, to identify the fungal genus using traditional mycological methods, it is necessary to carry out study for a longer period of time. The structures and spores of fungi which allow to identify them appear during 2–3 weeks of incubation or in some cases even after 30 days [44].

The potential of the described device to detect fungal contamination in indoor air was also verified by *in-situ* examinations on the buildings that differed in level of mould threat, supplemented with evaluation of clean and synthetic air [45].

The results of Pan et al. [46] indicated the electronic nose measurements were effective in discriminating the three fungal genera: *Botrytis* sp., *Penicillium* sp. and *Rhizopus* sp. in biological materials. For the above discussed research the authors utilized an e-nose with 12 metal-oxide sensors, forming two arrays in separate chambers. Each array was made up of 6 sensors, one of them utilized Figaro sensors (TGS2620, TGS2180, TGS825, TGS822, TGS2600, TGS2602), whereas the other one comprised Capteur sensors (CAP01, CAP03, CAP06, CAP07, CAP23, CAP25). The substrates for growing fungi included popular construction materials, such as plasterboard, chipboard, oriented strain board and wallpaper. The selected fungi genera were incubated on agar for 1 week period. Afterwards, the construction materials were contaminated with the above-mentioned fungi. The results of measurements obtained by means of the e-nose were subjected to PCA algorithms, which enabled to detect 4 fungi genera with 80–85% accuracy.

The difficulty in utilizing MVOCs as an indicator of the presence of fungi is the disparity of their metabolic products. Low concentration of these substances may also hinder the detection of MVOCs and consequently–the detection of contamination status [24]. The amount of MVOCs produced by microorganisms in the contaminated buildings ranges from 50 to 100 μg/m^3^, while in the clean building used for reference it approximated 10 μg/m^3^. Average concentration of MVOCs in 600 buildings analysed in the USA equals 27 μg/m^3^, whereas the highest value noted amounts to 2000 μg/m^3^. Although the detection limit of most sensors usually equals tenths of μg/m^3^, such amount might prove too low to detect individual (single) substances [24].

In the research presented in this article, the concentration levels of particular detected substances varied between 11 and 143 μg/m^3^. As far as determining the building contamination is concerned, individual VOCs do not constitute a clear indicator of material contamination. While some of them are more abundant than others, contamination is signified only through the elevation of several MVOCs above the detection limit [26]. Therefore, utilizing multi-sensor arrays which form the e-nose is efficient.

On the basis of the obtained results we can predicate that the volatile compounds in studied samples would be directly related to the particular fungi genus contaminating the materials. The results indicated that *Aspergillus* sp. and *Penicillium* sp. were distinguished by the e-nose, but not identified. Based on the flavour changes, the e-nose could detect the contamination of substrates, distinguish the control not-contaminated and contaminated samples, as well as discriminate some of samples contaminated by specific fungi. The response of e-nose sensors may potentially be useful for discrimination between the infected and uninfected materials but also for detection of certain fungi [45]. Comparing these readouts with the literature reports, our work confirmed the results of Kuske et al. [26, 39], that such fungi genera as *Penicillium aurantiogriseum*, *Penicillium chrysogenum* and *Cladosporium sphaerospermum* can be detected with high accuracy using an e-nose.

## Conclusions

According to the research on the air analysis performed by means of MOS-type sensors array on the headspace of building materials contaminated and non-contaminated by fungal isolates, following conclusions may be formulated:

1. The employed electronic nose with thin-film MOS-type sensors enables the detection of the differences among the examined samples. This provides a basis for further development of fungi detection technique and identification in construction materials.
2. The performed studies indicate that the method involving the use of an e-nose with MOS-type sensors enables distinguishing between the non-contaminated and contaminated samples, shortly fungal contamination occurs.
3. The most efficient fungi detection occurred in the third hour of growing, which was reflected by the notable differences between the contaminated and non-contaminated samples.
4. Changes in concentration of fungal metabolic products in time can be presented as trajectories on PCA diagrams. It was noticed that in the case of genera extracted from natural building composites (hemp-lime composites), an increase of metabolites moved the representing points of the e-nose readouts towards the negative values of PC1 axis.
5. In order to enable the identification of the genera of fungi with a satisfactory accuracy, at least 3 measurements should be performed in appropriate time intervals (after 12 hours of growing), and then the direction, sense and trajectory shape of changes of array signal in time need to be taken into consideration. Technically, it can be done by analyzing PCA graphs for individual strains during the experiment. However, the results obtained for all analyzed samples in individual hours of the experiment seem to be clearer and easier in interpretation.
6. The results of headspace analysis with chromatography method allow to distinguish the differences between particular samples contaminated with fungi after 72 hours. Similarly to e-nose readouts after 360 hours of fungal growth, the differences between the analysed samples become less differentiated–the concentration of detected VOC substances is decreasing.

## Supporting information

S1 Dataset Resistance readouts from the e-nose. (CSV)
